# Vitiligo as a Potential Overlooked Comorbidity in Schizophrenia: Insights From Two Case Reports

**DOI:** 10.7759/cureus.113727

**Published:** 2026-07-31

**Authors:** Amina Abdelhamid, Mohamed Amine Ennaciri, Siham B Belbachir

**Affiliations:** 1 Psychiatry, Arrazi University Psychiatric Hospital of Salé, Faculty of Medicine and Pharmacy of Rabat, Mohammed V University, Rabat, Salé, MAR; 2 Dermatology, Ibn Baja Hospital, Taza, MAR

**Keywords:** autoimmune disorders, phenothiazines, psychodermatology, schizophrenia, vitiligo

## Abstract

The co-occurrence of dermatological and psychiatric conditions has long been recognized, owing in part to the shared ectodermal origin of the skin and the nervous system. Vitiligo, an autoimmune depigmenting disorder, and schizophrenia, a chronic psychotic illness increasingly associated with immune dysregulation, represent two conditions whose potential relationship remains insufficiently explored in the literature.

We report two cases of male patients with long-standing schizophrenia who developed vitiligo over the course of their illness. The first patient, a 38-year-old man, presented with acrofacial vitiligo evolving over 11 years under long-acting injectable fluphenazine. The second patient, a 43-year-old man with a dark skin phototype, exhibited predominantly acral vitiligo with islands of repigmentation, evolving over 17 years, with exposure to haloperidol and chlorpromazine for 24 years. Both patients displayed classic schizophrenic symptomatology, including delusions, impaired judgment, and poor insight.

The association between vitiligo and schizophrenia is supported by epidemiological evidence, with vitiligo patients carrying a significantly elevated risk of developing schizophrenia. Several converging biological mechanisms may underlie this relationship, including shared immune dysregulation, oxidative stress, lipid abnormalities, and a common neuroectodermal origin. Additionally, phenothiazine-class antipsychotics - to which both patients were exposed - have been shown to exert cytotoxic effects on melanocytes and inhibit melanogenesis in vitro, suggesting that drug-induced depigmentation may represent an additional contributing factor in this population.

These cases illustrate the complex and potentially multifactorial relationship between vitiligo and schizophrenia. A holistic, interdisciplinary approach involving both dermatologists and psychiatrists is essential for the optimal management of patients presenting with both conditions. Further prospective and mechanistic studies are needed to better characterize the nature of this association and to identify potential shared therapeutic strategies.

## Introduction

Schizophrenia, a chronic and debilitating psychotic disorder, is frequently accompanied by a wide range of comorbidities, among which cutaneous manifestations are far from uncommon. It has been hypothesized that the co-occurrence of skin disorders in psychiatric patients may stem, at least in part, from the shared ectodermal origin of both the skin and the nervous system, a common embryological background that may underlie the well-documented bidirectional relationship between dermatological and mental health conditions [1].

Among the spectrum of skin disorders encountered in this context, vitiligo stands out as a condition of particular interest as a chronic autoimmune depigmenting skin disorder, and schizophrenia has been increasingly recognized for its associations with autoimmune diseases and linked to immunological alterations [2]. This intersection raises compelling questions about shared pathophysiological mechanisms and warrants closer clinical and scientific attention. These two case reports aim to open discussion on the various hypotheses that may account for a potential relationship between the two conditions, including the role of antipsychotic treatments.

## Case presentation

Case 1

A 38-year-old man was referred to our department for psychiatric evaluation. He had been under psychiatric follow-up for 21 years, with multiple hospitalizations throughout the course of his illness. His psychiatric history dated back 23 years, with an insidious onset in early adulthood. Notably, during the first two years following symptom onset, the patient did not seek medical attention, as his family attributed his symptoms to mystical causes and bewitchment, thereby delaying psychiatric care. He was subsequently initiated on an undocumented treatment, which he refused to take, as reported by the patient's family. Following this, he was transitioned to long-acting injectable fluphenazine decanoate, which he had been receiving for approximately 16 years. However, adherence to his therapeutic regimen remained consistently poor, driven by a combination of factors including a tendency toward wandering behavior, limited illness insight, and inadequate family involvement in supporting treatment compliance. As a result, the patient did not receive his injections regularly and was lost to follow-up on multiple occasions.

On mental status examination, the patient was calm and alert but poorly cooperative. He exhibited formal thought disorder with loosening of associations and poverty of content of speech. Prominent positive symptoms were noted, including unsystematized delusions - predominantly of a persecutory nature - and auditory hallucinations. Insight into his condition was severely impaired, and judgment was markedly compromised. These findings were consistent with a chronic, poorly controlled schizophrenia evolving over the past 23 years, according to the *Diagnostic and Statistical Manual of Mental Disorders, Fifth Edition, Text Revision *(DSM-5-TR) criteria [3].

Treatment was switched to risperidone 6 mg per day, with the intention of transitioning the patient to long-acting injectable paliperidone palmitate, resulting in good clinical improvement, critical appraisal of his delusional ideation, and regression of auditory hallucinations.

Regarding his dermatological history, a dermatologist-confirmed diagnosis of vitiligo, established with the aid of Wood's lamp examination, had been evolving for approximately 11 years. It was of the acrofacial type, characterized by well-demarcated, non-pruritic ivory-white macules symmetrically distributed over the perioral and paramandibular regions of the face, as well as the nasal bridge, eyelids, and auricles, without leukotrichia (Figure 1). Similar lesions were also present on the extremities, elbows, and knees. No mucosal involvement was observed. He denied any personal or family history of autoimmune or thyroid disease.

**Figure 1:**
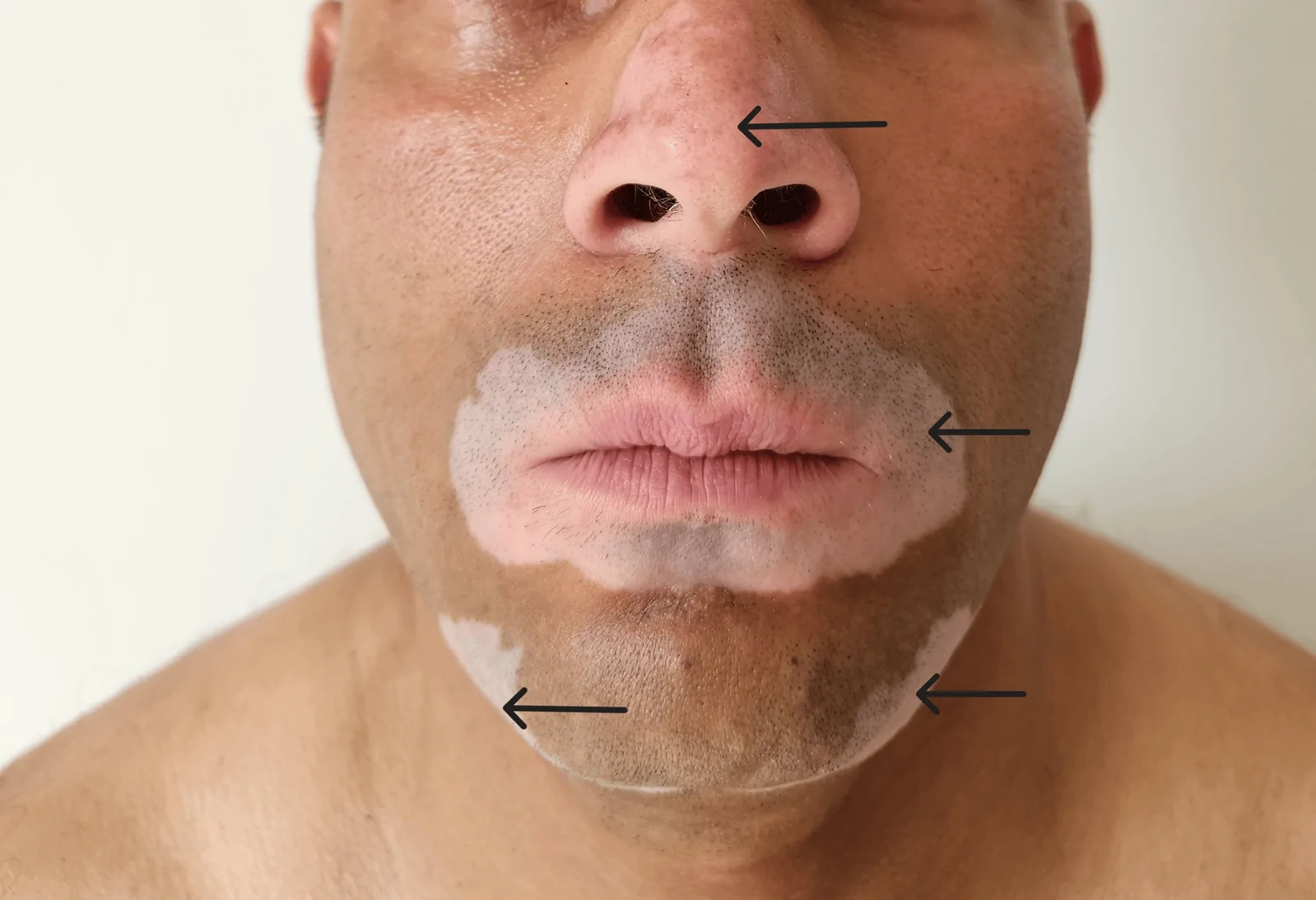
Facial symmetrical achromic macules in a patient with schizophrenia (Case 1)

Case 2

A 43-year-old man presented for psychiatric follow-up. He had been under psychiatric care for 24 years, with his first psychiatric hospitalization occurring 20 years ago and the last one 2 years ago, following which he was discharged on a combination of haloperidol and chlorpromazine. However, he discontinued both medications after a few months and was subsequently lost to follow-up for an extended period. His family history was notably significant, with two cousins diagnosed with psychotic disorders, suggesting a possible hereditary predisposition.

On mental status examination, the patient was alert but guarded and minimally collaborative. His speech was of normal rate and volume. He presented with prominent delusions, predominantly of a persecutory and mystico-religious nature, without overt hallucinations reported at the time of evaluation. Insight into his psychiatric condition was impaired, and judgment was markedly altered. His overall presentation was consistent with chronic schizophrenia evolving over approximately 27 years, with a course characterized by prolonged periods of untreated illness secondary to poor adherence and disengagement from psychiatric care.

The patient was hospitalized, restarted on the same treatment regimen, and discharged after one month following clinical improvement. He was additionally initiated on long-acting injectable haloperidol decanoate to ensure better therapeutic adherence.

From a dermatological standpoint, the patient had a dark skin phototype and presented with predominantly acral vitiligo confirmed by dermatologists upon Wood's lamp examination that had been evolving for approximately 17 years. The lesions were similar in appearance to those of the first patient; however, islands of repigmentation were also observed (Figure 2). He reported no personal or family history of autoimmune, thyroid, or other systemic conditions known to be associated with vitiligo.

**Figure 2:**
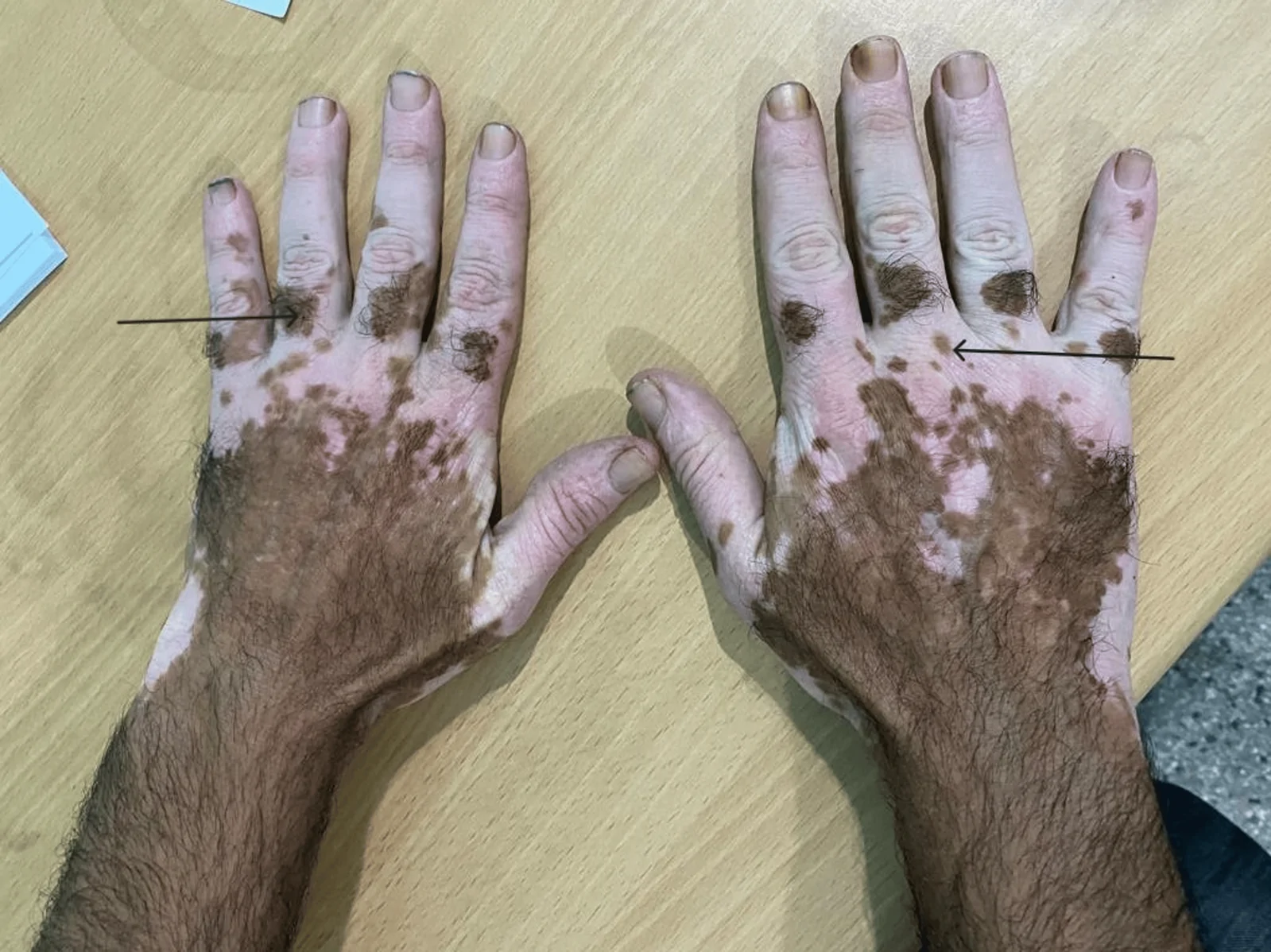
Acral symmetrical achromic macules with islands of repigmentation in a patient with schizophrenia (Case 2)

## Discussion

Vitiligo is an autoimmune condition estimated to affect around 0.2% of the general population, manifesting as depigmented macules and patches resulting from the loss or destruction of melanocytes within the epidermis [4]. Despite its recognized immune-mediated nature, the potential relationship between vitiligo and schizophrenia has received limited scientific attention, with only a small number of studies having examined this association to date.

Among the available evidence, a 2019 cross-sectional analysis on the National Inpatient Sample in the United States identified a significant association between vitiligo and psychiatric hospitalization for schizophrenia among other mental health disorders [5]. Consistent with these findings, a cross-sectional study conducted in a dermatology department in Jaipur involving 402 patients reported that approximately 3.77% of individuals with vitiligo were found to have comorbid schizophrenia [6]. A separate hospital-based investigation further noted that 16% of schizophrenic psychiatric patients had a concurrent pigmentary disorder [1].

A 2023 retrospective cohort analysis in the United States found that compared to a matched control cohort, patients with vitiligo showed a significantly elevated risk of developing both psychiatric and autoimmune comorbidities. Among the psychiatric conditions examined, the most pronounced associations were observed for schizophrenia, followed by social phobia and adjustment disorder, identifying these as the psychiatric conditions carrying the greatest relative risk in the vitiligo population [7].

Nonetheless, a 2024 pooled analysis combining data from cross-sectional and case-control studies demonstrated that individuals with vitiligo faced a significantly higher likelihood of experiencing depression, anxiety, and obsessive-compulsive disorder compared to matched controls. In contrast, no statistically significant associations were established with suicidal ideation, substance-related disorders, attention-deficit/hyperactivity disorder, schizophrenia, or adjustment disorder [8]. The absence of statistically significant association may reflect a lack of statistical power rather than a true absence of association, given the limited number of studies available.

On the other hand, it has been documented that psychotropic medication can lead to color changes affecting the skin, mucous membranes, hair, and nails in varying degrees. Such pigmentation alterations are especially frequent in patients undergoing treatment with phenothiazines, which both our patients are taking, with skin pigmentation anomalies identified in approximately 1.7% of 768 schizophrenic inpatients studied. Furthermore, vitiligo-like hypopigmented lesions have been described in some patients with schizophrenia treated with fluphenazine [9].

In fact, in a study about in vitro melanogenesis inhibition, both fluphenazine and prochlorperazine demonstrated concentration-dependent cytotoxic effects on normal human melanocytes. Beyond their impact on cell viability, both agents were found to suppress melanin synthesis and reduce tyrosinase activity at specific concentrations, an effect attributed to the direct inhibition of enzymatic activity [10].

In addition to neurocutaneous and embryological links, immune dysregulation and drug-induced depigmentation, oxidative stress and lipid dysregulation can also be considered as a possible common biological pathway. As fundamental constituents of cell membranes, lipids may play a significant role in the pathogenesis of both vitiligo and schizophrenia through their molecular diversity and capacity to engage in various chemical reactions. Multiple observations have linked both diseases to heightened stress responses, and research data highlight intricate interactions between oxidative stress and metabolic syndrome, of which lipid abnormalities represent a core feature in the context of both disorders. Preliminary clinical investigations suggest a potential benefit of statins in both vitiligo and schizophrenia, though their efficacy and therapeutic value in these settings warrant further investigation through larger and more rigorous studies [11].

## Conclusions

The two cases presented invite reflection on a potentially complex and multifaceted relationship between vitiligo and schizophrenia, two conditions whose co-occurrence may not be entirely coincidental, though this remains to be established. Both patients exhibited long-standing vitiligo alongside chronic schizophrenia, and notably, both were exposed to phenothiazine-class antipsychotics, raising the question of whether drug-induced melanocyte toxicity may have contributed to or exacerbated their depigmentation - a hypothesis that warrants further investigation.

Beyond the drug-induced hypothesis, several converging biological mechanisms have been proposed that could potentially underlie an association between vitiligo and schizophrenia, including their shared neuroectodermal origin, immune dysregulation, and oxidative stress-related lipid abnormalities. The existence of a true and significant comorbidity has yet to be confirmed by large-scale, well-designed studies.

We would also like to emphasize the importance of an interdisciplinary approach in the management of patients presenting with both vitiligo and schizophrenia, involving close collaboration between psychiatrists and dermatologists to ensure comprehensive and holistic patient care.
